# Utility of ChatGPT in Clinical Practice

**DOI:** 10.2196/48568

**Published:** 2023-06-28

**Authors:** Jialin Liu, Changyu Wang, Siru Liu

**Affiliations:** 1 Information Center West China Hospital Sichuan University Chengdu China; 2 Department of Medical Informatics West China Medical School Chengdu China; 3 Department of Otolaryngology-Head and Neck Surgery West China Hospital Sichuan University Chengdu China; 4 West China College of Stomatology Sichuan University Chengdu China; 5 Department of Biomedical Informatics Vanderbilt University Medical Center Nashville, TN United States

**Keywords:** ChatGPT, artificial intelligence, large language models, clinical practice, large language model, natural language processing, NLP, doctor-patient, patient-physician, communication, challenges, barriers, recommendations, guidance, guidelines, best practices, risks

## Abstract

ChatGPT is receiving increasing attention and has a variety of application scenarios in clinical practice. In clinical decision support, ChatGPT has been used to generate accurate differential diagnosis lists, support clinical decision-making, optimize clinical decision support, and provide insights for cancer screening decisions. In addition, ChatGPT has been used for intelligent question-answering to provide reliable information about diseases and medical queries. In terms of medical documentation, ChatGPT has proven effective in generating patient clinical letters, radiology reports, medical notes, and discharge summaries, improving efficiency and accuracy for health care providers. Future research directions include real-time monitoring and predictive analytics, precision medicine and personalized treatment, the role of ChatGPT in telemedicine and remote health care, and integration with existing health care systems. Overall, ChatGPT is a valuable tool that complements the expertise of health care providers and improves clinical decision-making and patient care. However, ChatGPT is a double-edged sword. We need to carefully consider and study the benefits and potential dangers of ChatGPT. In this viewpoint, we discuss recent advances in ChatGPT research in clinical practice and suggest possible risks and challenges of using ChatGPT in clinical practice. It will help guide and support future artificial intelligence research similar to ChatGPT in health.

## Introduction

ChatGPT is a large language model developed by OpenAI. It is based on the GPT architecture and uses deep learning techniques to generate natural language text [1,2]. The model has been developed using supervised and reinforcement learning strategies [3]. ChatGPT can generate coherent, grammatically correct text, which is an important development in artificial intelligence (AI) [4]. It shows great potential for using large language models and reinforcement learning from human feedback to improve clinical decision support (CDS) alert logic and potentially other medical areas involving complex clinical logic, a key step in the development of an advanced learning health care system. ChatGPT has quickly gained worldwide attention for its accurate well-formulated responses to various topics. As physicians, we have the opportunity to help guide and develop new ways of using this powerful tool. It can be used in research and development to analyze large amounts of medical data, identify trends, and provide insights into best clinical practices. Physicians need to consider using ChatGPT in their clinical practice. Furthermore, we are using ChatGPT as a tool to support physicians’ clinical practice, not to replace them.

Despite the increasing popularity and performance of ChatGPT, there is still a lack of studies evaluating its use in clinical practice. At the same time, we should be aware that ChatGPT is a double-edged sword, with powerful functions and potential dangers. To better understand the application of ChatGPT in clinical practice, we introduced the recent progress of ChatGPT in clinical practice to help interested researchers effectively grasp the key aspects of this topic and to provide possible future research directions. The purpose of this viewpoint is to provide an overview of the recent advances in ChatGPT in clinical practice (Multimedia Appendix 1 [5-16]), to explore the future direction of ChatGPT in clinical practice, to highlight the risks and challenges of its use in clinical practice, and to propose appropriate mitigation strategies. Although ChatGPT has demonstrated promising prospects in clinical practice, further research is needed to refine and improve its capabilities. Integrating ChatGPT into existing electronic health record (EHR) systems has the potential to improve diagnostic accuracy, treatment planning, and patient outcomes. However, it is essential to regard ChatGPT as a valuable tool that supplements the expertise of health care professionals rather than replacing them.

## Clinical Decision Support

Clinical decision-making is a complex process. It involves many factors, such as the physician’s clinical thinking, clinical reasoning, individual judgement, and the patient’s condition [17]. These factors can lead to cognitive biases, errors in reasoning, and preventable harm. AI-based CDS can effectively support physicians’ clinical decisions and improve treatment outcomes [18]. Current applications of ChatGPT in CDS include the following:

- Differential-diagnosis lists: Hirosawa et al [5] evaluated ChatGPT-3 and general internal medicine physicians to generate clinical cases, correct diagnoses, and five differential diagnoses for 10 common chief complaints. In the 10 differential diagnosis lists, the correct diagnosis rate of ChatGPT-3 was 28 out of 30 (93.3%). In the 5 differential diagnosis lists, the correct diagnosis rate of physicians was superior to ChatGPT-3 (98.3% vs 83.3%; *P*=.03). In the 10 differential diagnosis lists generated by ChatGPT-3, the consistent differential diagnosis rate of the doctors was 62 out of 88 (70.5%). This study shows that the differential diagnosis list generated by ChatGPT-3 has high diagnostic accuracy for clinical cases with common chief complaints.
- Clinical decision-making: Rao et al [6] entered all 36 published clinical vignettes from the Merck Sharp & Dohme (MSD) Clinical Manual into ChatGPT and compared the accuracy of differential diagnosis, diagnostic tests, final diagnosis, and management according to the patient age and gender, and the sensitivity of the case. ChatGPT achieved an overall accuracy rate of 71.7% (95% CI 69.3%-74.1%) across all 36 clinical cases.
- Cancer screening: Rao et al [7] compared ChatGPT responses with the American College of Radiology appropriateness criteria for breast pain and breast cancer screening. The ChatGPT prompt formats were open-ended (OE) and select all that apply (SATA). The results of the study showed that breast cancer screening achieved an average OE score of 1.83 out of 2, with an average correct rate of 88.9% for SATA; breast pain achieved an average OE score of 1.125 out of 2, with an average correct rate of 58.3% for SATA. The results show the feasibility of using ChatGPT for radiological decision-making and have the potential to improve clinical workflow.
- CDS optimization: Liu et al [8] studied 5 clinicians’ ratings of 36 CDS recommendations generated by ChatGPT and 29 recommendations generated by experts. The research results revealed that 9 of the top 20 recommendations in the survey were generated by ChatGPT. The study found that recommendations generated by ChatGPT provided a unique perspective and were rated as highly understandable and relevant, moderately useful but with low acceptability, bias, inversion, and redundancy. These recommendations can be an important complementary part of optimizing CDS alerts, identifying potential improvements to alert logic and supporting their implementation or even helping experts to develop their recommendations for CDS improvements.

ChatGPT has been evaluated for CDS applications. It has been shown to generate accurate lists of differential diagnoses, clinical decision making, optimize CDS, and provide insights for cancer screening decisions. Further research could focus on developing advanced models that integrate ChatGPT with existing CDS systems. These models can leverage the extensive medical literature, clinical guidelines, and patient data to support physicians in making accurate diagnoses, formulating treatment plans, and predicting patient outcomes. By combining the expertise of health care professionals with the capabilities of ChatGPT, comprehensive and personalized decision support is provided.

## Question-Answer (Medical Queries)

Intelligent question-answering is often used to provide information about diseases or to discuss the results of clinical tests. The use of intelligent question-answering in clinical practice has various benefits for health care systems, such as support for health care professionals and patients, triage, disease screening, health management, consultation, and training of health care professionals [19]. ChatGPT can be used for intelligent question-answering in health care. However, it should be noted that the answers may change over time and with different question prompts and that harmful biases in answers may occur [9]. It is important to use ChatGPT responsibly to ensure that they can help and not harm users seeking disease knowledge and information. Below are some examples of ChatGPT’s application in medical queries, demonstrating its potential in generating intelligent questions and answer prompts for various diseases:

- Common retinal diseases: Potapenko et al [10] conducted a study to evaluate the accuracy of ChatGPT in providing information on common retinal diseases: age-related macular degeneration, diabetic retinopathy, retinal vein occlusion, retinal artery occlusion, and central serous chorioretinopathy. A total of 100 responses were obtained through a series of questions that included the disease summary, prevention, treatment options, and prognosis for each disease. The results indicate that ChatGPT provides highly accurate general information (median score 5, IQR 4-5, range 3-5), disease prevention information (median 4, IQR 4-5, range 4-5), prognosis information (median 5, IQR 4-5, range 3-5), and treatment options (median 3, IQR 2-3, range 2-5). Reliability statistics showed a Cronbach α of .910 (95% CI .867-.940). Of the 100 responses evaluated, 45 were rated as very good with no inaccuracies, 26 had minor harmless inaccuracies, 17 were marked as potentially misinterpreted inaccuracies, and 12 had potentially harmful errors.
- Obstetrics and gynecology: Grünebaum et al [9] presented a series of questions (14 questions) on obstetrics and gynecology to ChatGPT, and evaluated the answers to each question. The study shows that ChatGPT is valuable for users seeking preliminary information on almost any topic in the field. The answers are generally convincing and informative. They do not contain a significant number of errors or misinformation. A major drawback is that the data on which the model is trained does not appear to be easily updatable.
- Hepatic disease: Yeo et al [11] investigated the accuracy and reproducibility of the ChatGPT in answering questions about knowledge, management, and emotional support for cirrhosis and hepatocellular carcinoma (HCC). The responses to the 164 questions in ChatGPT were independently assessed by two transplant hepatologists and reviewed by a third reviewer. The results of the study showed that ChatGPT had extensive knowledge of cirrhosis (79.1% correct) and HCC (74% correct). However, only a small proportion (47.3% for cirrhosis and 41.1% for HCC) was rated as comprehensive. Performance was better in basic knowledge, lifestyle, and treatment than in diagnosis and prevention. Regarding quality measures, the model answered 76.9% of questions correctly but failed to provide specific decision cutoff points and treatment duration. ChatGPT may have a role as a supplementary information tool for patients and physicians to improve outcomes.
- Cancer: Johnson et al [12] used questions from the “Common Cancer Myths and Misconceptions” web page to assess the accuracy of ChatGPT and National Cancer Institute (NCI) answers to the questions. The results showed an overall accuracy of 100% for NCI answers and 96.9% for questions 1 to 13 output by ChatGPT (k=–0.03, SE 0.08). There was no significant difference in word count and readability between NCI and ChatGPT answers. ChatGPT provided accurate information about common cancer myths and misconceptions.

The use of ChatGPT in answering medical queries has shown promise in assisting health care professionals by providing reliable information and guidance. However, ChatGPT’s responses are generated based on patterns and knowledge learned from training data, and it does not currently have up-to-date medical information or take into account specific patient situations. Therefore, health care providers should exercise caution and independently verify key information obtained from ChatGPT to ensure accuracy and appropriateness for individual patients. Careful and responsible use, as well as continued research and development, are necessary to maximize its benefits and minimize potential limitations.

## Medical Document

### Overview

Writing medical documents is a tedious and time-consuming process for health care providers. At the same time, errors in medical documentation are common [20,21]. Correctly documenting and exchanging clinical information between physician and patient is paramount. Medical documentation requires a high level of accuracy, so recorders should be able to capture and accurately record all medical information discussed during the interview. ChatGPT is an effective tool for medical documentation [13,22]. Using ChatGPT as a language assistant or providing templates can significantly reduce the time and improve the accuracy of medical documentation for clinicians [2]. The following four subsections illustrate specific areas where ChatGPT can be effectively applied, including the generation of patient clinic letters, radiology reports, medical notes, and discharge summaries, demonstrating its potential to simplify medical documentation and improve clinician efficiency.

### Patient Clinic Letters

Using skin cancer as an example, Ali et al [14] evaluated the readability, factual accuracy, and humanization of clinical letters to patients generated by ChatGPT. Of the 38 hypothetical clinical scenarios created, 7 involved basal cell carcinoma, 11 to squamous cell carcinoma, and 20 to malignant melanoma. The overall median accuracy of the clinical information in the letter was 7 (range 1-9). The overall median humanness of the writing style was 7 (5-9). The weighting for accuracy κ was 0.80 (*P*<.001) and for humanness 0.77 (*P*<.001). This assessment demonstrates that ChatGPT can generate clinical letters with high overall accuracy and humanization. In addition, the reading level of these letters is generally similar to that of letters currently generated by doctors.

### Radiology Reports

Jeblick et al [15] investigated 15 radiologists to assess the quality of the ChatGPT simplified radiology reports. Of all the ratings, 75% were “agree” or “strongly agree” (Q3=2) and none chose “strongly disagree.” Most radiologists felt that the simplified reports were accurate and complete and that there was no potential harm to patients. The initial results of this study indicate that there is great potential to use ChatGPT to improve patient-centered care in radiology.

### Medical Notes

ChatGPT helps doctors write medical notes. ChatGPT can write a structured medical note for a patient admitted to the intensive care unit, providing information about ongoing treatments, laboratory samples, blood gas analysis, and respiratory and hemodynamic parameters. ChatGPT can correctly group most parameters into their appropriate sections, even if they are only in abbreviated form without any information about their meaning [16].

### Discharge Summaries

Chintagunta et al [13] leveraged the variability in GPT-3 output by using ensembling and infusion of medical knowledge, enabling its use as an integral component of an effective low-shot learning method for medical summarization. GPT-3 takes as input a priming context for performing a task on a previously unseen example. Priming context refers to the textual description of a task and some demonstrations of task performance. These studies show that ChatGPT allows physicians to enter a brief description of the specific information to include, concepts to elaborate, and instructions to explain, and output a formal discharge summary in a matter of seconds (panel) [13,23]. ChatGPT can also improve the quality of the discharge summary itself [23].

## Future Research Directions

### Real-Time Monitoring and Predictive Analytics

Continuous monitoring of patient data, such as vital signs, laboratory results, and wearable device data, offers the opportunity for early detection of clinical deterioration and proactive intervention. Future research could explore how ChatGPT can analyze and interpret these real-time data streams, identifying patterns, trends, and abnormal changes. ChatGPT can provide timely alerts, risk assessment, and predictive analytics that enable health care professionals to intervene early and prevent adverse events when integrated into a monitoring system.

### Precision Medicine and Personalized Treatment

ChatGPT can analyze patient-specific data, including genetic information, biomarkers, and treatment history, to generate tailored treatment recommendations and predict individual responses to therapies. ChatGPT can help physicians and patients by analyzing complex data sets and generating personalized treatment recommendations. Further research could focus on developing ChatGPT models that use large-scale genomic and clinical data to provide more accurate predictions of treatment outcomes, identify optimal therapeutic approaches, and assist in clinical trial matching for precision medicine initiatives.

### Telemedicine and Remote Health Care

As telemedicine and telehealth continue to evolve, ChatGPT’s role in facilitating virtual patient-physician interactions could be explored. It may involve the development of ChatGPT-based virtual assistants that can assist health care professionals in triaging patients, providing initial assessments, and providing remote guidance for home care. In addition, ChatGPT could be trained to solve patient problems, provide health education, and support self-care at home. ChatGPT could play an important role in facilitating virtual doctor-patient interactions by providing virtual assistance and remote guidance.

### Integration With Existing Health Care Systems

Seamless integration of ChatGPT into existing clinical workflow and EHR systems is essential for its effective use in health care settings. Research should focus on developing standards and protocols for interoperability, data exchange, and secure communication between ChatGPT and EHR systems. It would enable the efficient use of ChatGPT in real-time CDS and documentation. ChatGPT can be used to improve the efficiency and accuracy of extracting information from unstructured clinical notes and EHRs. Future research can explore the integration of ChatGPT into EHR systems to enable intelligent data extraction, summarization, and analysis to support clinical research, quality improvement initiatives, and evidence-based practice.

## Possible Risks and Challenges of Using ChatGPT

Despite the excellent results of ChatGPT in evaluation studies of its use in clinical practice, the potential negative effects should not be underestimated, including privacy, ethics, bias, and discrimination. ChatGPT can lead to intentional and unintentional misuse of various applications [16]. While not all of the proposed fraudulent uses are unique to ChatGPT, what is impressive is the effective acceleration of ChatGPT in creating false evidence and material with a high degree of plausibility [16]. ChatGPT may create hallucinations or false information. “Hallucination” refers to the fact that the content generated by the model is not based on reality, creating a completely fabricated story or fact [24]. Another concern is that ChatGPT can reproduce the biases present in the data on which it is trained [16]. In the health care field, the accuracy of information is crucial, and the presence of errors or inaccuracies in information is terrifying. To ensure the safe and reliable use of ChatGPT, a rigorous human review process and human involvement in the workflow are essential. Adherence to relevant standards and criteria, such as accuracy, reliability, interpretability, explainability, and user acceptance benchmarks, is necessary. It is imperative to explore existing frameworks and guidelines for evaluating AI systems in health care, developed by regulatory bodies or professional organizations, and implement them for ChatGPT. Validating ChatGPT against these benchmarks is vital to guarantee its safety and effectiveness in clinical practice.

Security measures must be taken to safeguard patient information while using ChatGPT, including encryption, access control, secure data storage, and compliance with privacy regulations. Patient data used for training and fine-tuning ChatGPT should be anonymized to protect privacy, and obtaining patient consent for data use during ChatGPT’s development and deployment is of utmost importance. Efforts should be made to mitigate reidentification risks and prevent the linkage of deidentified patient data to identifiable information. Documenting and monitoring system activities are crucial for accountability and auditability. A proactive approach is necessary to ensure ongoing patient privacy and data protection. Compliance with relevant laws, regulations, and guidelines, such as data protection regulations, patient privacy laws, and health care regulations specific to AI technology, should be considered. The impact of compliance on ChatGPT’s clinical integration and the necessary steps to ensure adherence to legal and ethical standards should be addressed.

While ChatGPT shows promise and has the potential to revolutionize the clinical practice paradigm, several barriers hinder its clinical application. First, it lacks the medical expertise and background required to comprehend complex relationships between conditions and treatments, as it is not specifically designed to answer medical questions. Therefore, the quality of recommendations generated by ChatGPT needs assessment from a clinical expert perspective, considering short-term and long-term impacts on clinical outcomes [5,16]. Second, ChatGPT’s training data is outdated and limited to information available up to September 2021 [25]. Given the rapid evolution of medical research and advancements, the lack of the latest information may impact its usability in clinical practice. Keeping ChatGPT’s training data up-to-date while ensuring data accuracy is crucial to address this limitation and enhance its use in clinical settings. Third, ChatGPT currently relies on manual input of information, necessitating future iterations to enable the automatic extraction of data from EHRs without the need for manual entry. However, managing patient data in this context presents significant challenges, as strict regulations must be in place to ensure patient privacy and prevent information misuse. Therefore, meticulous data storage and access management are essential [16].

In conclusion, we must be cautious and proactive in addressing potential risks when using ChatGPT to ensure the safety and quality of patient care. With a clear understanding of the capabilities and limitations of ChatGPT, researchers and practitioners can effectively use the technology while avoiding any unintended consequences. Regardless of our attitude toward ChatGPT, the development of AI is unstoppable. The wisest course of action is to embrace it and use its capabilities to improve human health care.

## Appendix

Multimedia Appendix 1 ChatGPT in clinical practice.

## Abbreviations

AI: artificial intelligence
CDS: clinical decision support
EHR: electronic health record
HCC: hepatocellular carcinoma
MSD: Merck Sharp & Dohme
NCI: National Cancer Institute
OE: open-ended
SATA: select all that apply

## References

[1] Bhattacharya K, Bhattacharya A, Bhattacharya N, Yagnik Vd, Garg P, Kumar S (2023). ChatGPT in surgical practice—a new kid on the block. Indian J Surg.

[2] Brown TB, Mann B, Ryder N, Subbiah M, Kaplan J, Dhariwal P, Neelakantan A, Shyam P, Sastry G, Askell A, Agarwal S, Herbert-Voss A, Krueger G, Henighan T, Child R, Ramesh A, Ziegler DM, Wu J, Winter C, Hesse C, Chen M, Sigler E, Litwin M, Gray S, Chess B, Clark J, Berner C, McCandlish S, Radford A, Sutskever I, Amodei D Language models are few-shot learners. arXiv.

[3] (2022). Introducing ChatGPT. OpenAI.

[4] Sakib MSI (2023). What is ChatGPT?. ResearchGate.

[5] Hirosawa T, Harada Y, Yokose M, Sakamoto T, Kawamura R, Shimizu T (2023). Diagnostic accuracy of differential-diagnosis lists generated by Generative Pretrained Transformer 3 Chatbot for clinical vignettes with common chief complaints: a pilot study. Int J Environ Res Public Health.

[6] Rao A, Pang M, Kim J, Kamineni M, Lie W, Prasad AK, Landman A, Dreyer KJ, Succi MD Assessing the utility of ChatGPT throughout the entire clinical workflow. medRxiv.

[7] Rao A, Kim J, Kamineni M, Pang M, Lie W, Succi MD Evaluating ChatGPT as an adjunct for radiologic decision-making. medRxiv.

[8] Liu S, Wright AP, Patterson B, Wanderer JP, Turer RW, Nelson SD, McCoy AB, Sittig DF, Wright A Assessing the value of ChatGPT for clinical decision support optimization. medRxiv.

[9] Grünebaum A, Chervenak J, Pollet SL, Katz A, Chervenak FA (2023). The exciting potential for ChatGPT in obstetrics and gynecology. Am J Obstet Gynecol.

[10] Potapenko I, Boberg-Ans LC, Stormly Hansen M, Klefter ON, van Dijk EHC, Subhi Y (2023). Artificial intelligence-based chatbot patient information on common retinal diseases using ChatGPT. Acta Ophthalmol.

[11] Yeo YH, Samaan JS, Ng WH, Ting P, Trivedi H, Vipani A, Ayoub W, Yang JD, Liran O, Spiegel B, Kuo A (2023). Assessing the performance of ChatGPT in answering questions regarding cirrhosis and hepatocellular carcinoma. Clin Mol Hepatol.

[12] Johnson S, King A, Warner E, Aneja S, Kann BH, Bylund CL (2023). Using ChatGPT to evaluate cancer myths and misconceptions: artificial intelligence and cancer information. JNCI Cancer Spectr.

[13] Chintagunta B, Katariya N, Amatriain X, Kannan A (2021). Medically aware GPT-3 as a data generator for medical dialogue summarization. Proceedings of the Second Workshop on Natural Language Processing for Medical Conversations.

[14] Ali SR, Dobbs TD, Hutchings HA, Whitaker IS (2023). Using ChatGPT to write patient clinic letters. Lancet Digit Health.

[15] Jeblick K, Schachtner B, Dexl J, Mittermeier A, Stüber AT, Topalis J, Weber T, Wesp P, Sabel B, Ricke J, Ingrisch M ChatGPT makes medicine easy to swallow: an exploratory case study on simplified radiology reports. arXiv.

[16] Cascella M, Montomoli J, Bellini V, Bignami E (2023). Evaluating the feasibility of ChatGPT in healthcare: an analysis of multiple clinical and research scenarios. J Med Syst.

[17] Jialin L, Siru L (2019). Essential Clinical Informatics.

[18] Filiberto AC, Leeds IL, Loftus TJ (2021). Editorial: machine learning in clinical decision-making. Front Digit Health.

[19] Milne-Ives M, de Cock C, Lim E, Shehadeh MH, de Pennington N, Mole G, Normando E, Meinert E (2020). The effectiveness of artificial intelligence conversational agents in health care: systematic review. J Med Internet Res.

[20] Madadin M, Alhumam AS, Bushulaybi NA, Alotaibi AR, Aldakhil HA, Alghamdi AY, Al-Abdulwahab NK, Assiri SY, Alumair NA, Almulhim FA, Menezes RG (2019). Common errors in writing the cause of death certificate in the Middle East. J Forensic Leg Med.

[21] Rababa'h AM, Mardini A, Ababneh M, Rababa M, Hayajneh M (2022). Medication errors in Jordan: a systematic review. Int J Crit Illn Inj Sci.

[22] Joshi A, Katariya N, Amatriain X, Kannan A Dr. Summarize: global summarization of medical dialogue by exploiting local structures. arXiv.

[23] Patel SB, Lam K (2023). ChatGPT: the future of discharge summaries?. Lancet Digit Health.

[24] Shen Y, Heacock L, Elias J, Hentel KD, Reig B, Shih G, Moy L (2023). ChatGPT and other large language models are double-edged swords. Radiology.

[25] Vaishya R, Misra A, Vaish A (2023). ChatGPT: Is this version good for healthcare and research?. Diabetes Metab Syndr.

