# Supplementary material for: Utility of ChatGPT in Clinical Practice
Source: J Med Internet Res. 2023 Jun 28;25:e48568. doi: 10.2196/48568 (PMC10365580; doi:10.2196/48568)
Supplement: Multimedia Appendix 1 [file jmir_v25i1e48568_app1.docx]

Table1 ChatGPT in clinical practice

| Author | Study Directions | Study Objectives | GPT Usage | Results |
| --- | --- | --- | --- | --- |
| Hirosawa T, et al.[5] | Clinical Decision Support | Differential-diagnosis lists | ChatGPT-3 vs. Physicians | 10 differential-diagnosis lists: the rate of correct diagnosis by ChatGPT-3 was 28/30 (93.3%).  5 differential-diagnosis lists: the rate of correct diagnosis by physicians vs. by ChatGPT-3 (98.3% vs. 83.3%, p = 0.03).  The top diagnosis: the rate of correct diagnosis by physicians vs. by ChatGPT-3 (93.3% vs. 53.3%, p < 0.001). |
| Rao A, et al.[6] | Clinical Decision Support | Clinical decision making | ChatGPT with the Merck Sharpe & Dohme Clinical Manual | Accuracy overall across all 36 clinical vignettes: 71.7% (95% CI, 69.3% to 74.1%).  Accuracy in making a final diagnosis: 76.9% (95% CI, 67.8% to 86.1%)  Accuracy in making an initial differential diagnosis: 60.3% (95% CI, 54.2% to 66.6%). |
| Rao A, et al.[7] | Clinical Decision Support | Cancer screening | ChatGPT with the American College of Radiology (ACR) Appropriateness Criteria for breast pain and breast cancer screening | Breast cancer screening prompts: an average open-ended (OE) score of 1.83 (out of 2) and a select all that apply (SATA) average percentage correct of 88.9%.  Breast pain prompts: an average OE score of 1.125 (out of 2) and a SATA average percentage correct of 58.3%. |
| Sir L, et al.[8] | Clinical Decision Support | CDS Optimization | ChatGPT-generated suggestions vs. human-generated suggestions | The 20 suggestions that scored highest for 7 alerts: 9 generated by ChatGPT (36 AI-generated suggestions) vs. 11 generated by human (29 human-generated suggestions). |
| Potapenko I, et al.[10] | Question-answer (medical queries) | Common retinal diseases | ChatGPT in providing information on common retinal diseases | 100 responses evaluated: 45 very good with no inaccuracies; 26 minor harmless inaccuracies; 17 potentially misinterpreted inaccuracies, and 12 potentially harmful errors.  Highly accurate general information (median score: 5; interquartile range: 4-5; range: 3-5); Disease prevention information (median: 4; interquartile range: 4-5; range: 4-5); Prognosis information (median: 5; interquartile range: 4-5; range: 3-5); Treatment options (median: 3; interquartile range: 2-3; range: 2-5).  Cronbach's alpha of 0.910 (95% confidence interval: 0.867-0.940). |
| Grünebaum A, et al.[9] | Question-answer (medical queries) | Obstetrics and Gynecology | ChatGPT with 14 questions on obstetrics and gynaecology | ChatGPT can be valuable for users who want preliminary information about virtually any topic in the field. |
| Yeo YH, et al.[11] | Question-answer (medical queries) | Hepatic Disease | ChatGPT with 164 questions on cirrhosis and hepatocellular carcinoma (HCC) | ChatGPT regurgitated extensive knowledge of cirrhosis (79.1% correct) and HCC (74.0% correct), but only small proportions (47.3% in cirrhosis, 41.1% in HCC) were labeled as comprehensive.  Quality measures: the model answered 76.9% of questions correctly but failed to specify decision-making cut-offs and treatment durations. |
| Johnson SB, et al.[12] | Question-answer (medical queries) | Cancer | ChatGPT vs. National Cancer Institute (NCI) | There were few noticeable differences in the number of words or the readability of the answers from NCI or ChatGPT. |
| Ali SR, et al.[14] | Medical document | Patient clinic letters | ChatGPT-generated clinical letters to patients | The overall median accuracy of the clinical information in the letter was 7 (range 1-9).  The overall median humanness of writing style was 7 (5-9).  The weighting for accuracy κ was 0.80 (P<0.0001) and for humanness 0.77 (P<0.0001). |
| Jeblick K, et al.[15] | Medical document | Radiology reports | ChatGPT simplified radiology reports | 75% of 15 radiologists ratings were ‘agree’ or ‘strongly agree’ (Q3=2) and none chose ‘strongly disagree’. |
| Cascella, M et al.[16] | Medical document | Medical notes | ChatGPT wrote the medical notes | Structured medical notes for ICU patients, providing information on ongoing treatments, lab samples, blood gas analysis, and respiratory and hemodynamic parameters. |
| Chintagunta, B, et al.[13] | Medical document | Discharge summaries | GPT-3 as a Data Generator for Medical Dialogue Summarization | GPT-3 as the backbone and scale 210 human labeled examples to yield results comparable to using 6400 human labeled examples (~30x). |
